# The entropy solution of a reaction–diffusion equation on an unbounded domain

**DOI:** 10.1186/s13660-019-1956-3

**Published:** 2019-01-08

**Authors:** Huashui Zhan, Yongping Li

**Affiliations:** 1 School of Applied Mathematics, Xiamen University of Technology, Xiamen, China; 2 Fujian Engineering and Research Center of Rural Sewage Treatment and Water Safety, Xiamen, China

**Keywords:** 35L65, 35K85, 35R35, Reaction–diffusion problem, Unbounded domain, Partial boundary value condition, The entropy solution

## Abstract

The degenerate parabolic equations from the reaction–diffusion problems are considered on an unbounded domain $\varOmega\subset\mathbb {R}^{N}$. It is expected that only a partial boundary should be imposed the homogeneous boundary value, but how to give the analytic expression of this partial boundary seems very difficult. A new method, which is called the general characteristic function method, is introduced in this paper. By this new method, a reasonable analytic expression of the partial boundary value condition is found. Moreover, the stability of the entropy solutions is established based on this partial boundary value condition.

## Introduction

In the theory of water infiltration through porous media, Darcy’s linear relation

$$V=-K(\theta)\nabla\phi, $$

satisfactorily describes the flow conduction provided that the velocities are small, where *V* represents the seepage velocity of water, *θ* is the volumetric moisture content, $K(\theta)$ is the hydraulic conductivity and *ϕ* is the total potential, which can be expressed as the sum of a hydrostatic potential $\psi(\theta)$ and a gravitational potential *z*

$$\phi=\psi(\theta)+z. $$

But when the flow has large velocities, Darcy’s linear relation is invalid. In this case, in order to obtain a more accurate description of the flow, several nonlinear versions have been proposed. One of these versions is

$$V=-K(\theta)\nabla\phi. $$

Moreover, if infiltration takes place in a horizontal column of the medium, then the continuity equation has the form

$$\frac{\partial\theta}{\partial t}+\frac{\partial V}{\partial x}=0. $$

Thus, one obtains

1.1 $$ \frac{\partial\theta}{\partial t}=\frac{\partial}{\partial x}\bigl(D(\theta)\theta_{x} \bigr), $$

with $D(\theta)=K(\theta)\psi'(\theta)$.

If one considers the convection process additionally, then Eq. (1.1) can be generalized to the following equation:

1.2 $$ \frac{\partial u}{\partial t} =\Delta A(u)+\operatorname{div}\bigl(b(u,x,t)\bigr),\quad (x,t)\in Q_{T}=\varOmega\times (0,T), $$

where $\varOmega\subset\mathbb{R}^{N}$ is a smooth domain, $b(s,x,t)=\{ b^{i}(s,x,t)\}$ is a $C^{1}$ function and

$$A(u)= \int_{0}^{u}a(s)\,ds,\quad a(s)\geq0. $$

In fact, Eq. (1.2) comes from many reaction–diffusion problems and has been deeply investigated, one can refer to [1–3] and [4] for more details.

For the initial-boundary value problem of Eq. (1.2), since the equation has the parabolic–hyperbolic mixed type, the initial value condition

1.3 $$ u(x,0)=u_{0}(x), \quad x\in\varOmega, $$

is usually needed. But how to give a suitable boundary value condition becomes an interesting and challenging problem. To see that, let us consider the completely degenerate case, $A(u)\equiv0$, since boundary layer may appear, the solutions may not assume the given condition

1.4 $$ u(x,t)=0, \quad (x,t)\in\partial\varOmega\times(0,T), $$

at the boundary, otherwise the problem will be overdetermined. To solve the problem, in [5], the authors first gave an interpretation of the boundary condition (1.4) as an “entropy” inequality on *∂Ω*, which is the so-called BLN condition. Later, in [6], the author extended the result to the case $L^{\infty}$ data. He proposed that the boundary condition should be held in the integral form by introducing appropriate boundary entropy (i.e. entropy flux pairs). After the work of [6], many efforts have been focused on the strongly degenerate parabolic–hyperbolic equations

1.5 $$ \frac{\partial u}{\partial t} =\sum_{i=1}^{N} \frac{\partial}{\partial x_{i}} \biggl(a^{ij}(u,x,t)\frac{\partial u}{\partial x_{j}} \biggr)+ \operatorname{div}\bigl(b(u,x,t)\bigr), $$

which includes Eq. (1.2) as the isotropic diffusion case. In particular, the homogeneous boundary condition was considered in [7–9], and the nonhomogeneous boundary condition was considered in [10–14]. In all this work, the boundary condition is not directly shown as (1.4) in sense of the trace, but it is elegantly implicitly contained in a family of entropy inequalities (for example [10]), or is treated in a special weak sense such as [11]. Also, one can refer to [15, 16] for the latest progress in this direction.

If we still insist on the boundary value condition is imposed in the sense of the trace, then only a partial boundary condition,

1.6 $$ u(x,t)=0, \quad (x,t)\in \varSigma_{1}\times(0,T), $$

is required generally, where $\varSigma_{1}\subseteq\partial\varOmega$ is a relative open subset. This idea follows the theory of the second order differential equations with nonnegative characteristic form [17]. By this theory, if one wants to consider the boundary value problem of the equation

$$\sum_{r,s=1}^{N+1}a^{rs}(x) \frac{\partial^{2} u}{\partial x_{r}\, \partial x_{s}}+\sum_{r=1}^{N+1} b_{r}(x)\frac{\partial u}{\partial x_{r}}+c(x)u=f(x), \quad x\in \widetilde{\varOmega} \subset\mathbb{R}^{N+1}, $$

one only needs to give partial boundary condition

$$u(x)=0,\quad x\in \varSigma_{p}\subseteq\partial\varOmega, $$

where

$$\begin{aligned}& \varSigma_{p}=\sum_{2}\cup\sum _{3}, \\& \varSigma_{2}=\bigl\{ x\in\partial\widetilde{\varOmega}: a^{rs}n_{r}n_{s}=0, \bigl(b_{r}-a^{rs}_{x_{s}} \bigr)n_{r}< 0\bigr\} , \\& \varSigma_{3} =\bigl\{ x\in\partial\widetilde{\varOmega}: a^{rs}n_{s}n_{r}>0\bigr\} , \end{aligned}$$

and $\{n_{s}\}$ is the unit inner normal vector of *∂Ω̃*. Following this idea, in [18], the authors had given a suitable partial boundary value condition in the sense of the trace to Eq. (1.5), provided that

$$S_{1}\cap\overline{S_{2}}=\emptyset, $$

where

$$\begin{aligned}& S_{1}=\bigl\{ (x,t)\in\partial\varOmega\times[0,T]: a^{ij}(0, x,t)n_{i}n_{j}=0\bigr\} , \\& S_{2}=\bigl\{ (x,t)\in\partial\varOmega\times[0,T]: a^{ij}(0, x,t)n_{i}n_{j}>0\bigr\} . \end{aligned}$$

In [19], the author had considered Eq. (1.2) in the half space of $\mathbb{R}^{N}$ and shown that $\varSigma_{1}=\emptyset$ in (1.6) when $b(u,x,t)=b(u)$ and ${b^{N}}'(0)\geq0$. If the domain *Ω* is bounded, our previous papers [20, 21] had shown that $\varSigma_{1}$ also can be an empty set in some cases. Recently, the case that $\varSigma _{1}\subseteq\partial\varOmega$ is a subset has been studied in [22], where $\varSigma_{1}\subseteq\partial\varOmega$ is represented by the distance function $d(x)=\operatorname{dist}(x,\partial\varOmega)$.

In order to find a reasonable analytic expression $\varSigma_{1}$, we will summarize these results to give a general method, which is called the general characteristic function method in this paper. We first give a basic conception. Let $g(x)$ be a continuous nonnegative function on $\mathbb{R}^{N}$, satisfying

$$\varOmega=\bigl\{ x\in\mathbb{R}^{N}: g(x)>0\bigr\} ,\qquad \partial\varOmega= \bigl\{ x\in \mathbb{R}^{N}: g(x)=0\bigr\} , $$

and $g(x)=0$, $x\in\mathbb{R}^{N}\setminus\varOmega$. Assume that there is a small enough $\lambda_{0}>0$, such that $g(x)\in C^{2}(\overline{\varOmega_{\lambda}})$ for any $\lambda<\lambda_{0}$, where

$$\varOmega_{\lambda}=\bigl\{ x\in\varOmega: g(x)< \lambda\bigr\} . $$

If a function $g(x)$ satisfies these requirements, we can call it as a general characteristic function of *Ω*. For example, $\varOmega \subset\mathbb{R}^{N}$ is a bounded domain with an uniformly $C^{2}$ smooth boundary, then the distance function $d(x)$ is a general characteristic function of *Ω*. Certainly, in this case, $d^{2}(x)$ is another general characteristic function of *Ω*. In other words, the general characteristic function of *Ω* is not unique.

By the general characteristic function method it means that the part of the boundary $\varSigma_{1}$ appearing in (1.6) can be expressed by the general characteristic function if one chooses a suitable test function in the entropy solution inequality (see below, inequality (2.2)).

In addition, different from [10–14, 20, 21] and [15, 16], the domain $\varOmega\subset\mathbb{R}^{N}$ is an unbounded domain with an appropriately smooth boundary in this paper. The main innovation is that, after imposing some restrictions on $a(s)$ and $b^{i}(s,x,t)$, the part of the boundary $\varSigma_{1}$ appearing in (1.6) can be expressed as

1.7 $$ \varSigma_{1}=\varSigma_{g}=\bigl\{ x\in\partial\varOmega: \Delta g+ \vert \nabla g \vert \geq0\bigr\} . $$

Moreover, depending on the partial boundary value condition (1.6), the stability of the entropy solutions to Eq. (1.2) can be proved.

Naturally, since the general characteristic function *g* is not unique, if one chooses another general characteristic function $g_{1}(x)$, the partial boundary value $\varSigma_{g_{1}}$ may be different from the first one $\varSigma_{g}$. By this token, we can say the partial boundary value condition (1.6) is the best if the partial boundary $\varSigma_{1}$ in (1.6) satisfies

$$\varSigma_{1}\subseteq\varSigma_{g}, $$

for any general characteristic function $g(x)$ and we may conjecture that $\varSigma_{1}$ can be expressed as

1.8 $$ \varSigma_{1}=\bigcap_{g} \varSigma_{g}. $$

If there is a function $g_{0}$ such that

1.9 $$ \varSigma_{g_{0}}=\varSigma_{1}=\bigcap_{g} \varSigma_{g}, $$

then we can call $g_{0}$ the best general characteristic function. However, whether such a $g_{0}$ exists or not is difficult to clarify.

Moreover, the presented work is based on the Kruzkov doubling of variables trick to study the stability of the solutions. Let us give a brief explanation. First, we assume that $x\in[0,1]$ and consider the hyperbolic equation

1.10 $$ \frac{\partial u}{\partial t}=\frac{\partial B(u)}{\partial x}, $$

it is well known that even if $u_{0}(x)$ is smooth, the solution $u(x,t)$ may not be continuous. Let $\varGamma_{u}$ be the set of all jump points of $u\in \mathit{BV}(Q_{T})$, $(\gamma_{t}, \gamma_{x})$ be the normal of $\varGamma _{u}$ at $X=(x,t)$, $u^{+}(X)$, $u^{-}(X)$ be the approximate limits of *u* at $X\in\varGamma_{u}$ with respect to $(v,Y-X)>0$ and $(v,Y-X)<0$, respectively, and $\bar{u}=\frac {u^{+}+u^{-}}{2}$ be the symmetric mean value. Then $u(x,t)$ not only satisfies

1.11 $$ \iint_{Q_{T}} \biggl(u\frac{\partial\varphi}{\partial t}-B(u)\frac {\partial\varphi}{\partial x} \biggr)\,dx\,dt=0, $$

for any $\varphi\in C_{0}^{\infty}(Q_{T})$, but it also satisfies the “entropy condition”

1.12 $$ (\bar{u}-k)\gamma_{t}\leq\bigl(\overline{B(u)}-B(k)\bigr) \gamma_{x},\quad \forall (x,t)\in\varGamma_{u}, k\in \mathbb{R}, $$

Kruzkov [23] noticed that one can combine (1.11) with (1.12) and obtain the following inequality:

1.13 $$ \iint_{Q_{T}}\operatorname{sgn}(u-k) \biggl\{ (u-k) \frac{\partial\varphi }{\partial t}-\bigl(B(u)-B(k)\bigr)\frac{\partial\varphi}{\partial x} \biggr\} \,dx\,dt\geq o, $$

is true for all $0\leq\varphi\in\in C_{0}^{\infty}(Q_{T})$ and $k\in \mathbb{R}$. By this innovative discovery, the uniqueness of the entropy solution to Eq. (1.10) was proved in [23] by a skillful method-which is called the Kruzkov doubling of variables trick since then, and the corresponding weak solutions are called the entropy solutions of Eq. (1.10).

Secondly, for the parabolic–hyperbolic mixed type equation

1.14 $$ \frac{\partial u}{\partial t}=\frac{\partial^{2} A(u)}{\partial x^{2}}+\frac{\partial B(u)}{\partial x}, $$

if there exists an interior point in the set $\{s: a(s)=0\}$, then the solution $u(x,t)$ of Eq. (1.14) also may be discontinuous and must have a similar “entropy condition”. A very tricky problem lies in that such a similar “entropy condition” cannot be depicted as in (1.12). Vol’pert and Hudjaev [24] directly generalized (1.13) to the following inequality:

1.15 $$ \iint_{Q_{T}}\operatorname{sgn}(u-k) \biggl\{ (u-k) \frac{\partial\varphi }{\partial t}-\frac{\partial A(u)}{\partial x}\varphi_{x} -\bigl(B(u)-B(k)\bigr) \frac{\partial\varphi}{\partial x} \biggr\} \,dx\,dt\geq o, $$

and called $u(x,t)$ an entropy solution to Eq. (1.15) provided that $u\in \mathit{BV}(Q_{T})\cap L^{\infty}(Q_{T})$, $\frac{\partial A(u)}{\partial u}\in L^{2}_{\mathrm{loc}}(Q_{T})$ and satisfies (1.15). Based on this definition, by the fact

$$A'(s)=a(s)=0, \quad \text{if } s\in I\bigl(u^{r}(0,t), u^{l}(0,t)\bigr) $$

it was shown that, if *u* and *v* are two entropy solutions of the initial-boundary value problem to Eq. (1.14),

1.16 $$ \iint_{Q_{T}}\operatorname{sgn}(u-v) \biggl\{ (u-v) \frac{\partial\varphi }{\partial t}-\beta(u-v)\frac{\partial\varphi}{\partial x}-(w_{1}-w_{2}) \frac{\partial\varphi}{\partial x} \biggr\} \,dx\,dt\geq 0, $$

for any $0\leq\varphi\in C^{\infty}(\overline{Q_{T}})$, $\operatorname{supp}\varphi\subset[0,1]\times(0,T)$. Here, $w_{1}=\frac{\partial A(u_{1})}{\partial x}$, $w_{1}=\frac{\partial A(u_{2})}{\partial x}$, $\beta =\int_{0}^{1}B'(\lambda u_{1}+(1-\lambda)u_{2})\,d\lambda$. By (1.16) and a very painstaking calculation and skillful work, the uniqueness of the entropy solutions was proved by the Kruzkov doubling of variables trick, one can refer to [24] or [1, pp. 299–324] for the details.

Naturally, one may easily generalize the inequality (1.15) to the case when the spatial variable $x\in\varOmega\subset\mathbb{R}^{N}$, i.e.

1.17 $$ \frac{\partial u}{\partial t}=\Delta A(u)+\sum_{i=1}^{N} \frac{\partial b^{i}(u)}{\partial x_{i}}, $$

define the corresponding entropy solution inequality

1.18 $$ \iint_{Q_{T}}\operatorname{sgn}(u-k) \Biggl\{ (u-k) \frac{\partial\varphi }{\partial t}-\nabla A(u)\cdot\nabla\varphi -\sum _{i=1}^{N}\bigl(b^{i}(u)-b^{i}(k) \bigr)\frac{\partial\varphi}{\partial x_{i}} \Biggr\} \,dx\,dt\geq o, $$

and prove its existence. However, how to use the Kruzkov doubling of variables trick to prove the uniqueness of the entropy solution, if one still insists on using a similar technique as that in [24] or [1, pp. 299–324], becomes much more difficult. One can refer for the explanation to our previous work [25, Appendix 2] where we considered details.

For the Cauchy problem of Eq. (1.17) (or Eq. (1.2)), the essential improvements were made in [26–31] etc. around 2000. By introducing various kinds of the entropy solutions, in [26–31] etc., the authors had succeeded to prove the stability of the new kind of the entropy solutions. For the initial-boundary value problem, the essential improvements were made even later, one can refer to [10, 11] and [19] etc., this is due to the fact that, besides the new definition of the entropy solution as that in the Cauchy problem, how to give a suitable boundary value condition to ensure the well-posedness of Eq. (1.17) (or Eq. (1.2)) becomes a difficult problem; some details are given above in this paper. In this paper, we will consider the initial-boundary value problem of Eq. (1.2) in an unbounded domain, and we use some ideas of [31] and [19] to introduce a kind of the entropy solution of Eq. (1.2). By the weak convergent theorem (Lemma 3.1) and using the gradient estimation (Lemma 3.2), we can prove the existence of the entropy solution by the parabolical regularization method. Moreover, we will give a suitable partial boundary value condition (1.6) by the general characteristic function method, and we shall use the Kruzkov doubling of variables trick to prove the stability of the entropy solutions.

This paper is arranged as follows. In the first section, we have given the introduction. In the second section, we give the definition of the entropy solution and the main results. In the third section, the existence of the entropy solution is proved. In the fourth section, the stability of the entropy solutions is researched. In the fifth section, in order to show the part of the boundary $\varSigma_{g}$ changing along with the choice of *g*, some examples are given, and one can see that how to choose a suitable general characteristic function to pick out the partial boundary value condition (1.5) is important. At last, an application of the partial boundary value conditio is given, and some conclusions are summarized.

## The definition of the entropy solution inequality and the main results

It is well known that, since Eq. (1.2) is of hyperbolic–parabolic characteristic, only imposing the entropy conditions, the weak solution can be unique. We use some ideas from our previous work [19, 31] to define the entropy solutions in $\mathit{BV}_{\mathrm{loc}}(Q_{T})$.

For small $\eta >0$, we set

$$S_{\eta}(s)= \int_{0}^{s}h_{\eta}(\tau)\,d\tau, \qquad h_{\eta}(s)=\frac {2}{\eta} \biggl(1-\frac{| s|}{\eta } \biggr)_{+}. $$

Then $h_{\eta}(s)\in C(\mathbb{R})$, and

2.1 $$ \bigl\vert S_{\eta }(s) \bigr\vert \leq1;\qquad \lim_{\eta\rightarrow0}S_{\eta }(s)= \operatorname{sgn}s,\qquad \lim_{\eta\rightarrow 0}sh_{\eta}(s)=0. $$

### Definition 2.1

A function *u* is said to be the entropy solution of Eq. (1.2) with the initial value (1.3) and the partial boundary value (1.6), if:

1. *u* satisfies
  $$u\in \mathit{BV}_{\mathrm{loc}}(Q_{T})\cap L^{\infty}(Q_{T}), \qquad \frac{\partial }{\partial x_{i}} \int_{0}^{u}\sqrt{a(s)}\,ds\in L^{2}(Q_{T}). $$
2. For any $\varphi\in C_{0}^{2}(Q_{T})$, $\varphi\geq0$, for any $k\in \mathbb{R}$, for any small $\eta >0$, *u* satisfies
  2.2 $$\begin{aligned}& \iint_{Q_{T}} \biggl[I_{\eta}(u-k)\varphi_{t}-B_{\eta }^{i}(u,x,t,k) \varphi_{x_{i}}+A_{\eta}(u,k)\Delta\varphi \\& \quad {}-S_{\eta}^{\prime}(u-k) \biggl\vert \nabla \int_{0}^{u}\sqrt{a(s)}\,ds \biggr\vert ^{2}\varphi \biggr]\,dx\,dt \\& \quad {}- \iint_{Q_{T}} \int_{k}^{u}b^{i}_{x_{i}}(s,x,t)S'_{\eta}(s-k) \,ds\varphi \,dx\,dt\geq0. \end{aligned}$$
3. For any positive constant *R* large enough,
  2.3 $$ \lim_{t\rightarrow0} \int_{\varOmega_{R}} \bigl\vert u(x,t)-u_{0}(x) \bigr\vert \,dx=0, $$
  where $\varOmega_{R}=\{x\in\varOmega: |x|< R\}$.
4. The partial boundary value condition (1.6) is satisfied in the sense of trace.

Here, the inequality (2.2) is called the entropy solution inequality, $\mathit{BV}_{\mathrm{loc}}(Q_{T})$ is the locally *BV* function space [1], $b(u,x,t)=\{b^{i}(u,x,t)\}$, and

$$\begin{aligned}& B_{\eta}^{i}(u,x,t,k) = \int_{k}^{u}\frac{\partial b^{i}(s,x,t)}{\partial s}S_{\eta} (s-k) \,ds, \\& A_{\eta}(u,k) = \int_{k}^{u}a(s)S_{\eta}(s-k)\,ds, \qquad I_{\eta }(u-k)= \int_{0}^{u-k}S_{\eta}(s)\,ds. \end{aligned}$$

Since the domain *Ω* is unbounded, we use some techniques, as we had used in considering the Cauchy problem [31], to prove the existence of the entropy solutions. Throughout this paper, the initial value $u_{0}(x)\in L^{\infty}(\varOmega)\cap L^{1}(\varOmega)$.

### Theorem 2.2

*If* $A(s)\in C^{1}(\mathbb{R})$, $b^{i}(s,x,t)\in C^{1}(\mathbb{R}\times\overline{\varOmega}\times[0,T])$, *then the problem* (1.2)*–*(1.3)*–*(1.6) *has an entropy solution*.

The main aim of this paper is to study the stability of the entropy solutions.

### Theorem 2.3

*Suppose that* $A(s)$ *is* $C^{1}(\mathbb{R})$, $b^{i}(s,x,t)\in C^{1}(\mathbb{R}\times\overline{\varOmega}\times[0,T])$, $g(x)$ *is a general characteristic function of* *Ω*. *Let* $u(x,t)$ *and* $v(x,t)$ *be solutions of Eq*. (1.2) *with different initial values* $u_{0}(x)$ *and* $v_{0}(x)$, *respectively*, *and with the same partial homogeneous boundary value condition*

2.4 $$ \gamma u|_{\varSigma_{g}}=\gamma v|_{\varSigma_{g}}=0, $$

*where* *g* *is a general characteristic function of* *Ω*, *and* $\varSigma_{p}$ *has the form* (1.7). *If* *x* *is close to* *∂Ω*, $\Delta g+|\nabla g|$ *is bounded*, *and there is a constant* $\delta<0$ *such that*

2.5 $$ \biggl\vert \frac{\partial b^{i}(s, x,t)}{\partial s} \biggr\vert \leq(1-\delta )a(s), $$

*then*

2.6 $$ \int_{\varOmega} \bigl\vert u(x,t)-v(x,t) \bigr\vert \nu_{\delta}(x)\,dx\leq \int _{\varOmega} \bigl\vert u_{0}(x)-v_{0}(x) \bigr\vert \nu_{\delta}(x) \,dx. $$

*Here*

$$\nu_{\delta}(x)=e^{-\delta\sqrt{1+|x|^{2}}}, $$

*and* *δ* *is a small positive constant*.

The condition (2.5) seems not good enough. Since one always expects that convection term is independent of $a(s)$, Eq. (1.2) can be of the hyperbolic–parabolic mixed type. Fortunately, we can find another condition to take the place of (2.5), and we obtain the following theorem.

### Theorem 2.4

*Suppose that* $A(s)$ *is* $C^{1}(\mathbb{R})$, $b^{i}(s,x,t)\in C^{1}(\mathbb{R}\times\varOmega\times[0,T])$, $g(x)$ *is a general characteristic function of* *Ω*. *Let* $u(x,t)$ *and* $v(x,t)$ *be solutions of Eq*. (1.2) *with different initial values* $u_{0}(x)$ *and* $v_{0}(x)$, *respectively*, *and with the same partial boundary value condition* (2.4). *If* *x* *is close to* *∂Ω*, $\Delta g+|\nabla g|$ *is bounded*, *and*

2.7 $$ \bigl\vert b^{i}(\cdot,x,t) \bigr\vert \leq c g(x), $$

*then the stability of the entropy solutions* (2.6) *is true*.

### Remark 2.5

If condition (2.5) or condition (2.7) is not true, whether the part of the boundary $\varSigma_{g}$ appearing in (1.6) is true is unknown.

### Remark 2.6

The general characteristic function method introduced above can be used to any kinds of hyperbolic–parabolic mixed type equations to find a reasonable analytic expression of the partial boundary value condition (1.6), no matter the domain *Ω* is bounded or not. Certainly, for different domains, one should choose different general characteristic functions.

If the domain *Ω* is bounded, and we choose $g(x)=d(x)$, then Theorem 2.4 is just a version of Theorem 1.4 in [22]. However, since the domain considered in this paper is unbounded, the test function *φ* appearing in the entropy inequality (2.2) cannot be chosen as $d(x)$ itself, in virtue of that $d(x)$ is not integrable on *Ω* generally. Also, one can see that the test function chosen in [22] cannot be used in the entropy inequality (2.2) when the domain is unbounded.

## Proof of Theorem 2.2

### Lemma 3.1

([32])

*Assume that* $\varOmega\subset \mathbb{R}^{N}$ *is an open set and let* $f_{k},f\in L^{q}(\varOmega)$, *as* $k\rightarrow\infty$, $f_{k}\rightharpoonup f$ *weakly in* $L^{q}(\varOmega)$, $1\leq q<\infty$. *Then*

3.1 $$ \lim\inf_{k\rightarrow\infty} \| f_{k}\| _{L^{q}(\varOmega)}^{q} \geq\| f\|_{L^{q}(\varOmega )}^{q}. $$

Consider the following parabolically regularized equation:

3.2 $$ \frac{\partial u}{\partial t}=\Delta A(u)+\frac{1}{n} \Delta u+\operatorname{div} \bigl(b(u,x,t)\bigr),\quad (x,t)\in Q_{nT}=\varOmega_{n} \times(0,T), $$

with

3.3 $$\begin{aligned}& u(x,t)=0,\quad (x,t)\in\partial\varOmega_{n}\times(0,T), \end{aligned}$$

3.4 $$\begin{aligned}& u(x,0)=u_{0n}(x),\quad x\in\varOmega_{n}, \end{aligned}$$

where, for large enough *n*, $\varOmega_{n}=\{x\in\varOmega:|x|< n\}$, and $u_{0n}(x)\in C^{\infty}_{0}(\varOmega_{n})$ such that $u_{0n}(x)$ locally uniformly converges to $u_{0}(x)$.

From [1, 33], we know there are classical solutions $u_{n}\in C^{2}(\overline{Q_{nT}})\cap C^{3}(Q_{nT})$, and by the maximum principle,

3.5 $$ | u_{n}|\leq\| u_{0n}\|_{L^{\infty }}\leq\| u_{0}\|_{L^{\infty }}. $$

Moreover, similar to [19, 31], we can prove the following lemma, the details of the proof are omitted here.

### Lemma 3.2

*Let* $u_{n}(x,t)$ *be the solution of the problem* (3.2)*–*(3.3)*–*(3.4). *Then*

3.6 $$ \int_{\varOmega}|\operatorname{grad}u_{n}| \nu_{\sigma}(x)\,dx\leq c, $$

*where* $|\operatorname{grad}u|^{2}=\sum_{i=1}^{N}|\frac{\partial u}{\partial x_{i}}|^{2}+|\frac{\partial u}{\partial t}|^{2}$, *c* *is independent of* *n*, *and*

$$\nu_{\sigma}(x)=e^{-\sigma\sqrt{1+|x|^{2}}}, $$

*σ* *is a given positive constant*.

By (3.2) and (3.6), we have

3.7 $$ \int_{0}^{T} \int_{\varOmega_{n}} \biggl(a(u_{n})+\frac{1}{n} \biggr) |\nabla u_{n}|^{2}\,dx\,dt\leq c. $$

Now, let

$$\bar{u}_{n}= \textstyle\begin{cases} u_{n}, & \mbox{if } x\in\varOmega_{n}, \\ 0, & \mbox{if } x\in\varOmega\setminus\varOmega_{n}. \end{cases} $$

Then, by (3.6) and (3.7), we have

3.8 $$\begin{aligned}& \int_{\varOmega}|\operatorname{grad}\bar{u_{n}}| \nu_{\sigma}(x)\,dx\,dt\leq c, \end{aligned}$$

3.9 $$\begin{aligned}& \int_{0}^{T} \int _{\varOmega} \biggl(a(\bar{u_{n}})+\frac {1}{n} \biggr) |\nabla \bar{u_{n}}|^{2}\nu_{\sigma}(x)\,dx \,dt\leq c. \end{aligned}$$

Thus there exists a subsequence $\{u_{{n}}\}$ of $\{\bar{u_{n}}\}$ and a function $u\in \mathit{BV}_{\mathrm{loc}}(Q_{T})\cap L^{\infty }(Q_{T})$ such that $u_{{n}}\rightarrow u$ a.e. on $Q_{T}$.

### Proof of Theorem 2.2

First of all, by (3.8), $u\in \mathit{BV}_{\mathrm{loc}}(Q_{T})$ and we can define the trace on the boundary, the partial boundary value condition is understood in the sense of the trace. Secondly, by (3.9)

3.10 $$\begin{aligned}& \frac{\partial}{\partial x_{i}} \int_{0}^{u_{n} }\sqrt{a(s)} \,ds\rightharpoonup \frac{\partial}{\partial x_{i}} \int_{0}^{u}\sqrt{a(s)}\,ds \quad \text{weakly in }L^{2}\bigl(\varOmega_{R}\times(0,T)\bigr),\forall R>0, \end{aligned}$$

3.11 $$\begin{aligned}& \frac{\partial}{\partial x_{i}} \int_{0}^{u}\sqrt{a(s)}\,ds\in L^{2} \bigl(\varOmega_{R}\times (0,T)\bigr),\quad \forall R>0, i=1,2,\ldots, N, \end{aligned}$$

where $\varOmega_{R}=\{x\in\varOmega:|x|< R\}$.

Moreover, let $\varphi\geq0$, $\varphi\in C_{0}^{2}(Q_{T})$, and multiply (3.2) by $\varphi S_{\eta}(u_{n}-k)$, and integrate over $Q_{T}$. Then we have

3.12 $$\begin{aligned}& \iint_{Q_{T}}\frac{\partial u_{n}}{\partial t}\varphi S_{\eta}(u_{n }-k) \,dx\,dt \\& \quad = \iint_{Q_{T}}\Delta A(u_{n})\varphi S_{\eta}(u_{n}-k)\,dx\,dt \\& \qquad {}+\frac{1}{n} \iint_{Q_{T}}\Delta u_{n}\varphi S_{\eta}(u_{n}-k) \,dx\,dt \\& \qquad {} +\sum_{i=1}^{N} \iint_{Q_{T}}\frac{\partial b^{i}(u_{n},x,t)}{\partial x_{i}}\varphi S_{\eta }(u_{n}-k) \,dx\,dt. \end{aligned}$$

Integration by part yields

3.13 $$\begin{aligned}& \iint_{Q_{nT}}I_{\eta}(u_{n}-k)\varphi _{t}\,dx\,dt+ \iint_{Q_{T}}A_{\eta}(u_{n},k)\triangle\varphi \,dx\,dt+ \iint_{Q_{T}}B_{\eta}^{i}(u_{n},x,t,k) \varphi _{x_{i}}\,dx\,dt \\& \qquad {}-\frac{1}{n} \iint_{Q_{T}}\nabla u_{n}\cdot\nabla\varphi S_{\eta}(u_{n }-k)\,dx\,dt-\frac{1}{n} \iint_{Q_{T}}|\nabla u_{n}|^{2}S_{\eta}^{\prime}(u_{n }-k) \varphi \,dx\,dt \\& \qquad {}+ \iint_{Q_{T}} \int_{k}^{u_{n}}b_{x_{i}}^{i}S_{\eta}(s-k) \,ds\varphi \,dx\,dt- \iint _{Q_{T}}a(u_{n})|\nabla u_{n}|^{2}S_{\eta}^{\prime}(u_{n }-k) \varphi \,dx\,dt \\& \quad =0. \end{aligned}$$

Since

3.14 $$\begin{aligned}& \lim_{n\rightarrow\infty}\frac{1}{n} \iint _{Q_{T}}\nabla u_{n}\cdot\nabla\varphi S_{\eta}(u_{n }-k)\,dx\,dt=0, \end{aligned}$$

3.15 $$\begin{aligned}& -\frac{1}{n} \iint _{Q_{T}}|\nabla u_{n}|^{2}S_{\eta}^{\prime}(u_{n }-k) \varphi \,dx\,dt\leq0, \end{aligned}$$

and by Lemma 3.1,

3.16 $$\begin{aligned}& \lim\inf_{n\rightarrow\infty} \iint_{Q_{T}}S_{\eta}^{\prime}(u_{n }-k)a(u_{n}) \frac{\partial u_{n}}{\partial x_{i}}\frac{\partial u_{n}}{\partial x_{i}}\varphi \,dx\,dt \\& \quad \geq \iint_{Q_{T}}S_{\eta}^{\prime}(u-k) \biggl\vert \nabla \int_{0}^{u}\sqrt{a(s)}\,ds \biggr\vert ^{2}\varphi \,dx\,dt, \end{aligned}$$

then letting $n \rightarrow\infty$ in (3.13), we can obtain (2.2).

At last, the initial value is true in the sense of (2.3) as can be shown in a similar way to that in [1, 31]. Then we have proved the existence of the entropy solution. □

## Proof of Theorem 2.3 and Theorem 2.4

### Proof of Theorem 2.3

Let $u(x,t)$, $v(x,t)$ be two entropy solutions of (1.2) with different initial values

4.1 $$ u(x,0)=u_{0}(x),\qquad v(x,0)=v_{0}(x), $$

and with the same partially boundary value condition

4.2 $$ u(x,t)=v(x,t)=0,\quad (x,t)\in\varSigma_{g}\times(0, T). $$

Here *g* is a general characteristic function of *Ω*, $\varSigma_{g}$ has the form of (1.7).

For any $\eta>0$, $k,l\in \mathbb{R}$, for any $0\leq\varphi\in C_{0}^{2}(Q_{T})$, by Definition 2.1

4.3 $$\begin{aligned}& \iint_{Q_{T}} \biggl\{ I_{\eta}(u-k)\varphi_{t}-B_{\eta }^{i}(u,x,t,k) \varphi_{x_{i}}+A_{\eta}(u,k)\Delta\varphi \\& \qquad {}+ \int_{k}^{u}b^{i}_{x_{i}}(s,x,t)S_{\eta}(s-k) \,ds\varphi-S_{\eta }^{\prime}(u-k) \biggl\vert \nabla \int_{0}^{u}\sqrt{a(s)}\,ds \biggr\vert ^{2}\varphi \biggr\} \,dx\,dt \\& \quad \geq0, \end{aligned}$$

4.4 $$\begin{aligned}& \iint_{Q_{T}} \biggl\{ I_{\eta}(v-l)\varphi_{\tau}-B_{\eta }^{i}(v,y, \tau, l)\varphi_{y_{i}}+A_{\eta}(v,l)\Delta\varphi \\& \qquad {}+ \int_{l}^{v}b^{i}_{y_{i}}(s,y, \tau)S_{\eta}(s-l)\,ds\varphi-S_{\eta }^{\prime}(v-l) \biggl\vert \nabla \int_{0}^{v}\sqrt{a(s)}\,ds \biggr\vert ^{2}\varphi \biggr\} \,dy\,d\tau \\& \quad \geq0 . \end{aligned}$$

As usual, we let $\psi(x,t,y,\tau)=\phi(x,t)j_{h}(x-y,t-\tau)$. Here $\phi (x,t)\geq0$, $\phi(x,t)\in C_{0}^{\infty}(Q_{T})$, $\omega_{h}$ is the mollifier and

4.5 $$ j_{h}(x-y,t-\tau)=\omega_{h}(t-\tau)\prod _{i=1}^{N}\omega _{h}(x_{i}-y_{i}). $$

If we choose $k=v(y,\tau)$, $l=u(x,t)$, $\varphi=\psi(x,t,y,\tau)$ in (4.3) (4.4), then, by the Kruzkov doubling of variables trick, we have

4.6 $$\begin{aligned}& \iint_{Q_{T}} \iint _{Q_{T}}I_{\eta }(u-v) (\psi_{t}+ \psi_{\tau}) \,dx\,dt\,dy\,d\tau \\& \qquad {}- \iint_{Q_{T}} \iint _{Q_{T}}\bigl[B_{\eta}^{i}(u,x,t,v)\psi _{x_{i}}+B_{\eta }^{i}(v,y, \tau, u)\psi_{y_{i}} \bigr]\,dx\,dt\,dy\,d\tau \\& \qquad {}+ \iint_{Q_{T}} \iint_{Q_{T}}\bigl[A_{\eta}(u,v)\Delta_{x} \psi+A_{\eta }(v,u)\Delta_{y}\psi\bigr] \,dx\,dt\,dy\,d\tau \\& \qquad {}+ \iint_{Q_{T}} \iint_{Q_{T}} \biggl[ \int _{v}^{u}b^{i}_{x_{i}}(s,x,t)S_{\eta}(s-v) \,ds\psi \\& \qquad {}+ \int _{u}^{v}b^{i}_{y_{i}}(s,y, \tau)S_{\eta}(s-u)\,ds\psi \biggr] \,dx\,dt\,dy\,d\tau \\& \qquad {}- \iint_{Q_{T}} \iint_{Q_{T}}S_{\eta}^{\prime}(u-v) \biggl( \biggl\vert \nabla \int_{0}^{u}\sqrt{a(s)}\,ds \biggr\vert ^{2}+ \biggl\vert \nabla \int_{0}^{v}\sqrt{a(s)}\,ds \biggr\vert ^{2} \biggr)\psi \,dx\,dt\,dy\,d\tau \\& \quad \geq0. \end{aligned}$$

Let $\eta\rightarrow0$, $h\rightarrow 0$ in (4.6). Then we obtain

4.7 $$\begin{aligned}& \iint_{Q_{T}} \bigl[ \bigl\vert u(x,t)-v(x,t) \bigr\vert \phi_{t}-\operatorname{sgn} (u-v) \bigl(b^{i}(u,x,t)-b^{i}(v,x,t) \bigr)\phi_{x_{i}} \\& \qquad {}+ \bigl\vert A(u)-A(v) \bigr\vert \Delta\phi \bigr] \,dx\,dt \\& \qquad {}+ \iint_{Q_{T}}\bigl[b^{i}_{x_{i}}(u,x,t)-b^{i}_{x_{i}}(v,x,t) \bigr]\operatorname{sgn}(u-v)\phi \,dx\,dt \\& \quad \geq0. \end{aligned}$$

For $0<\tau<s<T$, we denote

$$\eta(t)= \int_{\tau-t}^{s-t}\alpha_{\epsilon}(\sigma)\,d\sigma, \quad \epsilon< \min\{\tau, T-s\}, $$

where $\alpha_{\epsilon}(t)$ is the kernel of mollifier with $\alpha_{\epsilon}(t)=0$ for $t\notin(-\epsilon,\epsilon )$. Let us choose the test function

4.8 $$ \phi=\eta(t)\xi(x), $$

in (4.7), in which $\xi(x)\in C_{0}^{\infty}(\varOmega)$.

4.9 $$\begin{aligned}& \int_{\varOmega} \bigl\vert u(x,s)-v(x,s) \bigr\vert \xi(x)\,dx- \int_{\varOmega} \bigl\vert u(x,\tau )-v(x,\tau) \bigr\vert \xi(x) \,dx \\& \quad \leq \int_{s}^{\tau} \int_{\varOmega}\bigl[-\operatorname{sgn} (u-v) \bigl(b^{i}(u,x,t)-b^{i}(v,x,t) \bigr)\xi_{x_{i}} \\& \qquad {}+\bigl(b^{i}_{x_{i}}(u,x,t)-b^{i}_{x_{i}}(v,x,t) \bigr)\operatorname{sgn}(u-v)\xi (x)\bigr]\,dx\,dt \\& \qquad {}+ \int_{s}^{\tau} \int_{\varOmega} \bigl\vert A(u)-A(v) \bigr\vert \Delta\xi \,dx \,dt. \end{aligned}$$

Let

$$\nu_{\delta}=e^{-\delta\sqrt{1+|x|^{2}}}, $$

as before. By the limit process, we can choose *ξ* in (4.9) as

4.10 $$ \xi(x,t)=\omega_{\lambda}(x)\nu_{\delta}, $$

where $\omega_{\lambda}(x)\in C_{0}^{2}(\varOmega)$ is defined as follows: for any given small enough $0<\lambda$, $0\leq\omega_{\lambda}\leq1$, $\omega|_{\partial \varOmega}=0$ and

4.11 $$ \omega_{\lambda}(x)=1,\quad \mbox{if }g(x)\geq\lambda, $$

when $0\leq g(x)\leq\lambda$,

4.12 $$ \omega_{\lambda}\bigl(g(x)\bigr)=1-\frac{(g(x)-\lambda)^{2}}{\lambda ^{2}}. $$

Now,

4.13 $$\begin{aligned}& \omega_{\lambda x_{i}}= \textstyle\begin{cases} -\frac{2(g(x)-\lambda)}{\lambda^{2}}g_{x_{i}}, & \mbox{if } g(x)< \lambda, \\ 0, & \mbox{if } g(x)\geq{\lambda}, \end{cases}\displaystyle \end{aligned}$$

4.14 $$\begin{aligned}& \begin{aligned}[b] \Delta\bigl(\omega_{\lambda}\bigl(g(x)\bigr)\bigr) &= \nabla\bigl(\omega'_{\lambda}(g)\nabla g\bigr) \\ &=\omega''_{\lambda}(g)|\nabla g|^{2}+ \omega'_{\lambda}(g)\Delta g \\ &=-\frac{2|\nabla g|^{2}}{\lambda^{2}}-\frac{2(g(x)-\lambda)}{\lambda ^{2}}\Delta g, \quad \text{if } g(x)< \lambda, \end{aligned} \end{aligned}$$

and when $g(x)\geq\lambda$, $\Delta(\omega_{\lambda}(g(x)))=0$ clearly.

Since

$$\begin{aligned}& \nu_{\delta x_{i}}=-\delta\nu_{\delta}\frac{x_{i}}{\sqrt{1+|x|^{2}}}, \\& \nu_{\delta x_{i}x_{i}}=\delta^{2}\nu_{\delta}\frac {x_{i}^{2}}{1+|x|^{2}}- \delta\nu_{\delta}\frac{1+\sum_{j=1, j\neq i}^{N}|x_{j}|^{2}}{(1+|x|^{2})^{\frac{3}{2}}}, \end{aligned}$$

we have

4.15 $$\begin{aligned} \xi_{x_{i}}&=\nu_{\delta x_{i}}\omega_{\lambda}\bigl(g(x)\bigr)+ \nu_{\delta }\omega_{\lambda}'(g)g_{x_{i}}(x) \\ &=-\delta\nu_{\delta}\frac{x_{i}\omega_{\lambda}(g(x))}{\sqrt {1+|x|^{2}}}+\nu_{\delta} \omega'_{\lambda}(g)g_{x_{i}}(x) \\ &=-\delta\nu_{\delta}\frac{x_{i}\omega_{\lambda}(g(x))}{\sqrt {1+|x|^{2}}}-\nu_{\delta} \frac{2(g(x)-\lambda)}{\lambda ^{2}}g_{x_{i}}(x), \quad \text{if } g(x)< \lambda, \end{aligned}$$

while

4.16 $$ \xi_{x_{i}}=-\delta\nu_{\delta}\frac{x_{i}\omega_{\lambda }(g(x))}{\sqrt{1+|x|^{2}}},\quad \text{if } g(x)\geq\lambda. $$

Then

4.17 $$\begin{aligned} \Delta\xi&=\Delta\nu_{\delta}\omega_{\lambda}\bigl(g(x)\bigr)+2 \nu_{\delta x_{i}}\omega_{\lambda}'(g)g_{x_{i}}+ \nu_{\delta}\Delta\omega_{\lambda}\bigl(g(x)\bigr) \\ &=\biggl[\delta^{2}\nu_{\delta}\frac{|x|^{2}}{1+|x|^{2}}-\delta \nu_{\delta }\frac{N+(N-1)|x|^{2}}{(1+|x|^{2})^{\frac{3}{2}}}\biggr]\omega_{\lambda}\bigl(g(x)\bigr) \\ &\quad {}+4\delta\nu_{\delta}\frac{x_{i}}{\sqrt{1+|x|^{2}}}\frac{(g(x)-\lambda )}{\lambda^{2}}g_{x_{i}}(x) \\ &\quad {}-\nu_{\delta}\frac{2|\nabla g|^{2}}{\lambda^{2}}-\nu_{\delta} \frac {2(g(x)-\lambda)}{\lambda^{2}}\Delta g,\quad \text{if } g(x)< \lambda, \end{aligned}$$

and

4.18 $$ \Delta\xi=\biggl[\delta^{2}\nu_{\delta}\frac{|x|^{2}}{1+|x|^{2}}-\delta \nu _{\delta}\frac{N+(N-1)|x|^{2}}{(1+|x|^{2})^{\frac{3}{2}}}\biggr]\omega _{\lambda}\bigl(g(x) \bigr), \quad \text{if } g(x)\geq\lambda. $$

Accordingly,

4.19 $$\begin{aligned}& \int_{s}^{\tau} \int_{\varOmega} \bigl\vert A(u)-A(v) \bigr\vert \Delta\xi \,dx \,dt \\& \quad = \int_{s}^{\tau} \int_{\varOmega} \biggl[\delta^{2}\nu_{\delta} \frac {|x|^{2}}{1+|x|^{2}} -\delta\nu_{\delta}\frac{N+(N-1)|x|^{2}}{(1+|x|^{2})^{\frac {3}{2}}} \biggr] \omega_{\lambda}\bigl(g(x)\bigr)|A(u)-A(v)|\,dx\,dt \\& \qquad {}+4 \int_{s}^{\tau} \int_{\varOmega_{\lambda}}\delta\nu_{\delta}\frac {x_{i}}{\sqrt{1+|x|^{2}}} \frac{(g(x)-\lambda)}{\lambda ^{2}}g_{x_{i}}(x) \bigl\vert A(u)-A(v) \bigr\vert \,dx\,dt \\& \qquad {}- \int_{s}^{\tau} \int_{\varOmega\lambda} \biggl[\nu_{\delta}\frac {2|\nabla g|^{2}}{\lambda^{2}}+ \nu_{\delta}\frac{2(g(x)-\lambda )}{\lambda^{2}}\Delta g \biggr] \bigl\vert A(u)-A(v) \bigr\vert \,dx\,dt. \end{aligned}$$

At the same time,

4.20 $$\begin{aligned}& \int_{s}^{\tau} \int_{\varOmega} \bigl[-\operatorname{sgn} (u-v) \bigl(b^{i}(u,x,t)-b^{i}(v,x,t) \bigr)\xi_{x_{i}} \bigr]\,dx\,dt \\& \quad =\delta \int_{s}^{\tau} \int_{\varOmega} \bigl[\operatorname{sgn} (u-v) \bigl(b^{i}(u,x,t)-b^{i}(v,x,t) \bigr) \bigr]\nu_{\delta}\frac{x_{i}\omega _{\lambda}(g(x))}{\sqrt{1+|x|^{2}}}\,dx\,dt \\& \qquad {}+ \int_{s}^{\tau} \int_{\varOmega_{\lambda}} \bigl[\operatorname{sgn} (u-v) \bigl(b^{i}(u,x,t)-b^{i}(v,x,t) \bigr) \bigr]\nu_{\delta}\frac {2(g(x)-\lambda)}{\lambda^{2}}g_{x_{i}}(x)\,dx\,dt. \end{aligned}$$

Once more

4.21 $$\begin{aligned}& \int_{s}^{\tau} \int_{\varOmega}\bigl[b^{i}_{x_{i}}(u,x,t)-b^{i}_{x_{i}}(v,x,t) \bigr]\operatorname{sgn}(u-v)\xi(x)\,dx\,dt \\& \quad = \int_{s}^{\tau} \int_{\varOmega}\bigl[b^{i}_{x_{i}}(u,x,t)-b^{i}_{x_{i}}(v,x,t) \bigr]\operatorname{sgn}(u-v)\omega_{\lambda}(x)\nu_{\delta}\,dx \,dt. \end{aligned}$$

By (4.9), (4.19)–(4.21), and using the assumption

$$\biggl\vert \frac{\partial{b^{i}}(s,x,t)}{\partial s} \biggr\vert \leq(1-\delta ) a(s), $$

by the Cauchy mean value theorem, we have

4.22 $$\begin{aligned}& \int_{\varOmega} \bigl\vert u(x,s)-v(x,s) \bigr\vert \xi(x)\,dx- \int_{\varOmega} \bigl\vert u(x,\tau )-v(x,\tau) \bigr\vert \xi(x) \,dx \\ & \quad \leq \int_{s}^{\tau} \int_{\varOmega}\bigl[-\operatorname{sgn} (u-v) \bigl(b^{i}(u,x,t)-b^{i}(v,x,t) \bigr)\xi_{x_{i}} \\ & \qquad{}+\bigl(b^{i}_{x_{i}}(u,x,t)-b^{i}_{x_{i}}(v,x,t) \bigr)\operatorname{sgn}(u-v)\xi (x)\bigr]\,dx\,dt \\ & \qquad{}+ \int_{s}^{\tau} \int_{\varOmega} \bigl\vert A(u)-A(v) \bigr\vert \Delta\xi \,dx \,dt \\ & \quad \leq \int_{s}^{\tau} \int_{\varOmega_{\lambda}}\nu_{\delta } \bigl\vert A(u)-A(v) \bigr\vert \frac{-2(g(x)-\lambda)}{\lambda^{2}} \\ & \qquad{}\cdot \biggl[\Delta g -\delta\frac{2x_{i}g_{x_{i}}}{\sqrt{1+ \vert x \vert ^{2}}} -\frac{\operatorname{sgn} (u-v)(b^{i}(u,x,t)-b^{i}(v,x,t))}{ \vert A(u)-A(v) \vert }g_{x_{i}}(x) \biggr]\,dx\,dt \\ & \qquad{}+c \int_{s}^{\tau} \int_{\varOmega}\nu_{\delta} \bigl\vert u(x,t)-v(x,t) \bigr\vert \,dx\,dt \\ & \quad \leq \int_{s}^{\tau} \int_{\varOmega_{\lambda}}\nu_{\delta } \bigl\vert A(u)-A(v) \bigr\vert \frac{-2(g(x)-\lambda)}{\lambda^{2}} \\ & \qquad {}\cdot\biggl[\Delta g + \biggl(\delta\frac{2 \vert x_{i} \vert }{\sqrt{1+ \vert x \vert ^{2}}}+ \frac{ \vert {b^{i}}'(\zeta ,x,t) \vert }{a(\zeta)} \biggr) \bigl\vert g_{x_{i}}(x) \bigr\vert \biggr] \,dx\,dt \\ & \qquad{}+c \int_{s}^{\tau} \int_{\varOmega}\nu_{\delta} \bigl\vert u(x,t)-v(x,t) \bigr\vert \,dx\,dt \\ & \quad \leq \int_{s}^{\tau} \int_{\varOmega_{\lambda}}\nu_{\delta } \bigl\vert A(u)-A(v) \bigr\vert \frac{-2(g(x)-\lambda)}{\lambda^{2}}\bigl[\Delta g +(\delta+1-\delta) \bigl\vert g_{x_{i}}(x) \bigr\vert \bigr]\,dx\,dt \\ & \qquad{}+c \int_{s}^{\tau} \int_{\varOmega}\nu_{\delta} \bigl\vert u(x,t)-v(x,t) \bigr\vert \,dx\,dt \\ & \quad \leq \int_{s}^{\tau} \int_{\varOmega_{\lambda}}\nu_{\delta } \bigl\vert A(u)-A(v) \bigr\vert \frac{-2(g(x)-\lambda)}{\lambda^{2}}\bigl[\Delta g + \vert \nabla g \vert \bigr]\,dx\,dt \\ & \qquad{}+c \int_{s}^{\tau} \int_{\varOmega}\nu_{\delta} \bigl\vert u(x,t)-v(x,t) \bigr\vert \,dx\,dt \\ & \leq \int_{s}^{\tau} \int_{\varOmega_{\lambda1}}\nu_{\delta } \bigl\vert A(u)-A(v) \bigr\vert \frac{-2(g(x)-\lambda)}{\lambda^{2}}\bigl[\Delta g + \vert \nabla g \vert \bigr]\,dx\,dt \\ & \qquad{} +c \int_{s}^{\tau} \int_{\varOmega}\nu_{\delta} \bigl\vert u(x,t)-v(x,t) \bigr\vert \,dx\,dt. \end{aligned}$$

Here, we have denoted

$$\varOmega_{\lambda1}=\bigl\{ x\in\varOmega_{\lambda}: \Delta g + \vert \nabla g \vert \geq0\bigr\} $$

and

$${b^{i}}'(\zeta, x,t)=\frac{\partial{b^{i}}(s,x,t)}{\partial s} \bigg|_{s=\zeta}. $$

By the definition of the trace, we have

4.23 $$\begin{aligned}& \lim_{\lambda\rightarrow0} \int_{\varOmega_{\lambda1}}\nu_{\delta } \bigl\vert A(u)-A(v) \bigr\vert \frac{-2(g(x)-\lambda)}{\lambda^{2}}\bigl[\Delta g + \vert \nabla g \vert \bigr]\,dx \\& \quad = \int_{\varSigma_{p}}\nu_{\delta}\bigl[\Delta g + \vert \nabla g \vert \bigr] \bigl\vert A(u)-A(v) \bigr\vert \, d\varSigma \\& \quad =0. \end{aligned}$$

Let $\lambda\rightarrow0$ in (4.20). By (4.21), we have

4.24 $$\begin{aligned}& \int_{\varOmega} \bigl\vert u(x,s)-v(x,s) \bigr\vert \nu_{\delta}(x)\,dx \\& \quad \leq \int_{\varOmega} \bigl\vert u(x,\tau)-v(x,\tau) \bigr\vert \nu_{\delta}\,dx+c \int _{s}^{\tau} \int_{\varOmega}\nu_{\delta} \vert u-v \vert \,dx \,dt. \end{aligned}$$

By the Gronwall inequality, we have the conclusion. □

### Proof of Theorem 2.4

Similar to the proof of Theorem 2.3, the calculations (4.1)–(4.21) are still true. In addition, by the assumption of (2.7), we have

4.25 $$\begin{aligned}& \int_{\varOmega} \bigl\vert u(x,s)-v(x,s) \bigr\vert \xi(x)\,dx- \int_{\varOmega} \bigl\vert u(x,\tau )-v(x,\tau) \bigr\vert \xi(x) \,dx \\& \quad \leq \int_{s}^{\tau} \int_{\varOmega}\bigl[-\operatorname{sgn} (u-v) \bigl(b^{i}(u,x,t)-b^{i}(v,x,t) \bigr)\xi_{x_{i}} \\& \qquad {}+\bigl(b^{i}_{x_{i}}(u,x,t)-b^{i}_{x_{i}}(v,x,t) \bigr)\operatorname{sgn}(u-v)\xi (x)\bigr]\,dx\,dt \\& \qquad {}+ \int_{s}^{\tau} \int_{\varOmega} \bigl\vert A(u)-A(v) \bigr\vert \Delta\xi \,dx \,dt \\& \quad \leq \int_{s}^{\tau} \int_{\varOmega_{\lambda}}\nu_{\delta } \bigl\vert A(u)-A(v) \bigr\vert \frac{-2(g(x)-\lambda)}{\lambda^{2}} \biggl[\Delta g -\delta\frac{2x_{i}g_{x_{i}}}{\sqrt{1+ \vert x \vert ^{2}}} \biggr]\,dx \,dt \\& \qquad {}+ \int_{s}^{\tau} \int_{\varOmega_{\lambda}}\nu_{\delta}\frac {2(g(x)-\lambda)}{\lambda^{2}} \operatorname{sgn} (u-v)\bigl[b^{i}(u,x,t)-b^{i}(v,x,t) \bigr]g_{x_{i}}(x)\,dx\,dt \\& \qquad {}+c \int_{s}^{\tau} \int_{\varOmega}\nu_{\delta} \bigl\vert u(x,t)-v(x,t) \bigr\vert \,dx\,dt \\& \quad \leq \int_{s}^{\tau} \int_{\varOmega_{\lambda}}\nu_{\delta } \bigl\vert A(u)-A(v) \bigr\vert \frac{-2(g(x)-\lambda)}{\lambda^{2}}\bigl[\Delta g +\delta \vert \nabla g \vert \bigr] \,dx\,dt \\& \qquad {}+c \int_{s}^{\tau} \int_{\varOmega_{\lambda}}\nu_{\delta}\frac {1}{\lambda}g(x) \vert \nabla g \vert \,dx\,dt \\& \qquad {}+c \int_{s}^{\tau} \int_{\varOmega}\nu_{\delta} \bigl\vert u(x,t)-v(x,t) \bigr\vert \,dx\,dt. \end{aligned}$$

Since $x\in\varOmega_{\lambda}$, $g(x)\leq\lambda$,

4.26 $$ \lim_{\lambda\rightarrow0} \int_{\varOmega_{\lambda}}\nu_{\delta }\frac{1}{\lambda}g(x)|\nabla g| \,dx=0. $$

By (4.25) and (4.26), similar the proof of Theorem 2.3, we have the conclusion. □

From the proof of Theorem 2.3 and Theorem 2.4, we easily obtain the following theorem.

### Theorem 4.1

*Suppose that* $A(s)$ *is a* $C^{1}$ *function*, $b^{i}(s,x,t)$ *is a* $C^{1}$ *function on* $\mathbb{R}\times\overline{\varOmega}\times [0,T]$, $g(x)$ *is a general characteristic function of* *Ω*. *Let* $u(x,t)$, $v(x,t)$ *be solutions of Eq*. (1.2) *with different initial values* $u_{0}(x)$, $v_{0}(x)$, *respectively*. *If there is a general characteristic function of* *Ω* *such that*

4.27 $$\begin{aligned}& \bigl\vert b^{i}(\cdot,x,t) \bigr\vert \leq c g(x), \end{aligned}$$

4.28 $$\begin{aligned}& \Delta g\leq0,\qquad \vec{x}\cdot\nabla g\geq0, \end{aligned}$$

*then*

4.29 $$ \int_{\varOmega} \bigl\vert u(x,t)-v(x,t) \bigr\vert \nu_{\delta}(x)\,dx\leq \int _{\varOmega} \bigl\vert u_{0}(x)-v_{0}(x) \bigr\vert \nu_{\delta}(x) \,dx. $$

### Proof

First, we still have (4.1)–(4.18). Secondly, by (4.28),

4.30 $$\begin{aligned}& \int_{s}^{\tau} \int_{\varOmega} \bigl\vert A(u)-A(v) \bigr\vert \Delta\xi \,dx \,dt \\& \quad = \int_{s}^{\tau} \int_{\varOmega}\biggl[\delta^{2}\nu_{\delta} \frac{ \vert x \vert ^{2}}{1+ \vert x \vert ^{2}} -\delta\nu_{\delta}\frac{N+(N-1) \vert x \vert ^{2}}{(1+ \vert x \vert ^{2})^{\frac {3}{2}}}\biggr] \omega_{\lambda}\bigl(g(x)\bigr) \bigl\vert A(u)-A(v) \bigr\vert \,dx\,dt \\& \qquad {}+4 \int_{s}^{\tau} \int_{\varOmega_{\lambda}}\delta\nu_{\delta}\frac {x_{i}}{\sqrt{1+ \vert x \vert ^{2}}} \frac{(g(x)-\lambda)}{\lambda ^{2}}g_{x_{i}}(x) \bigl\vert A(u)-A(v) \bigr\vert \,dx\,dt \\& \qquad {}- \int_{s}^{\tau} \int_{\varOmega\lambda} \biggl[\nu_{\delta}\frac {2 \vert \nabla g \vert ^{2}}{\lambda^{2}}+ \nu_{\delta}\frac{2(g(x)-\lambda )}{\lambda^{2}}\Delta g \biggr] \bigl\vert A(u)-A(v) \bigr\vert \,dx\,dt \\& \quad \leq \int_{s}^{\tau} \int_{\varOmega}\biggl[\delta^{2}\nu_{\delta} \frac { \vert x \vert ^{2}}{1+ \vert x \vert ^{2}} -\delta\nu_{\delta}\frac{N+(N-1) \vert x \vert ^{2}}{(1+ \vert x \vert ^{2})^{\frac {3}{2}}}\biggr] \omega_{\lambda}\bigl(g(x)\bigr) \bigl\vert A(u)-A(v) \bigr\vert \,dx\,dt \\& \quad \leq c \int_{s}^{\tau} \int_{\varOmega}\nu_{\delta} \bigl\vert u(x,t)-v(x,t) \bigr\vert \,dx\,dt \end{aligned}$$

and by (4.27)

4.31 $$\begin{aligned}& \lim_{\lambda\rightarrow0} \int_{s}^{\tau} \int_{\varOmega} \bigl[-\operatorname{sgn} (u-v) \bigl(b^{i}(u,x,t)-b^{i}(v,x,t) \bigr)\xi_{x_{i}} \bigr]\,dx\,dt \\& \quad =\lim_{\lambda\rightarrow0}\delta \int_{s}^{\tau} \int_{\varOmega } \bigl[\operatorname{sgn} (u-v) \bigl(b^{i}(u,x,t)-b6{i}(v,x,t) \bigr) \bigr]\nu_{\delta}\frac{x_{i}\omega _{\lambda}(g(x))}{\sqrt{1+ \vert x \vert ^{2}}}\,dx\,dt \\& \qquad {}+\lim_{\lambda\rightarrow0} \int_{s}^{\tau} \int_{\varOmega_{\lambda }} \bigl[\operatorname{sgn} (u-v) \bigl(b^{i}(u,x,t)-b^{i}(v,x,t) \bigr) \bigr]\nu_{\delta}\frac {2(g(x)-\lambda)}{\lambda^{2}}g_{x_{i}}(x)\,dx\,dt \\& \quad \leq c \int_{s}^{\tau} \int_{\varOmega}\nu_{\delta} \bigl\vert u(x,t)-v(x,t) \bigr\vert \,dx\,dt \\& \qquad {}+\lim_{\lambda\rightarrow0} \int_{s}^{\tau} \int_{\varOmega_{\lambda }} \bigl[\operatorname{sgn} (u-v) \bigl(b^{i}(u,x,t)-b^{i}(v,x,t) \bigr) \bigr]\nu_{\delta}\frac {2(g(x)-\lambda)}{\lambda^{2}}g_{x_{i}}(x)\,dx\,dt \\& \quad \leq c \int_{s}^{\tau} \int_{\varOmega}\nu_{\delta} \bigl\vert u(x,t)-v(x,t) \bigr\vert \,dx\,dt. \end{aligned}$$

Here, we have used the fact that

$$\begin{aligned}& \lim_{\lambda\rightarrow0} \biggl\vert \int_{s}^{\tau} \int_{\varOmega_{\lambda }} \bigl[\operatorname{sgn} (u-v) \bigl(b^{i}(u,x,t)-b^{i}(v,x,t) \bigr) \bigr]\nu_{\delta}\frac {2(g(x)-\lambda)}{\lambda^{2}}g_{x_{i}}(x)\,dx\,dt \biggr\vert \\& \quad \leq\lim_{\lambda\rightarrow0} \int_{s}^{\tau} \int_{\varOmega _{\lambda}}\frac{g^{2}(x)}{\lambda^{2}}\,dx\,dt \\& \quad =0. \end{aligned}$$

Thirdly,

4.32 $$\begin{aligned}& \biggl\vert \int_{s}^{\tau} \int_{\varOmega }\bigl[b^{i}_{x_{i}}(u,x,t)-b^{i}_{x_{i}}(v,x,t) \bigr]\operatorname{sgn}(u-v)\xi(x)\,dx\,dt \biggr\vert \\& \quad = \biggl\vert \int_{s}^{\tau} \int_{\varOmega }\bigl[b^{i}_{x_{i}}(u,x,t)-b^{i}_{x_{i}}(v,x,t) \bigr]\operatorname{sgn}(u-v)\omega_{\lambda }(x)\nu_{\delta}\,dx\,dt \biggr\vert \\& \quad \leq c \int_{s}^{\tau} \int_{\varOmega}\nu_{\delta} \bigl\vert u(x,t)-v(x,t) \bigr\vert \,dx\,dt. \end{aligned}$$

By (4.30)–(4.32), we have (4.24). Then we have the conclusion. □

Theorem 4.1 implies that, in some cases, one can obtain the stability (4.30) without the boundary value condition. In other words, the conditions (4.27)–(4.28) can take the place of the boundary value condition.

## Examples of the partial boundary value

In this section, we will give some examples to show that the part of the boundary $\varSigma_{g}$ in (1.7) changes along with the choice of the general characteristic function *g*.

(i) As we have said above, the general characteristic function method also can be used in the case that domain *Ω* is bounded. Let us first give an example to show, for the bounded domain, how the part of the boundary $\varSigma_{g}$ in (1.7) changes along with the choice of the general characteristic function *g*. Let $r>0$ be a given constant, and

$$\varOmega_{r}= \bigl\{ x\in\mathbb{R}^{2}: |x|^{2}>r^{2} \bigr\} . $$

If we choose

$$g_{1}(x)=|x|^{2}-r^{2}, $$

then

$$g_{1x_{i}}=2x_{i},\quad i=1,2;\qquad \Delta g_{1}=4. $$

On $\partial\varOmega_{r}$, $|x|=r$, we have

$$\Delta g_{1}+|\nabla g_{1}|=4+2r>0. $$

Then

5.1 $$ \varSigma_{g_{1}}=\partial\varOmega. $$

If we choose

$$g_{r}(x)=\ln\bigl(1+ \vert x \vert ^{2}-r^{2} \bigr), $$

then

$$\begin{aligned}& g_{rx_{i}}=\frac{2x_{i}}{1+|x|^{2}-r^{2}},\quad i=1,2, \\& \Delta g_{r}=\frac{2(-|x|^{2}+1-r^{2})}{1+|x|^{2}-r^{2}}. \end{aligned}$$

Then on $\partial\varOmega_{r}$,

5.2 $$ \varSigma_{g_{r}}= \bigl\{ x\in\partial\varOmega_{r}:\Delta g_{r}+|\nabla g_{r}|=2\bigl(1-2r^{2}+r\bigr)\geq0 \bigr\} . $$

Thus, if $r< 1$, then

5.3 $$ \varSigma_{g_{r}}=\emptyset, $$

which implies that there is no boundary value condition (1.5) required. In other words, when $r<1$, $g_{r}$ is the best characteristic function.

While $r\geq1$

5.4 $$ \varSigma_{g_{r}}=\partial\varOmega_{r}, $$

is the whole boundary and the boundary value condition (1.6) becomes the usual Dirichlet boundary value condition.

(ii) Two examples of the unbounded *∂Ω* are given in what follows. The first one is

$$\varOmega_{2}= \bigl\{ x\in\mathbb{R}^{2}: x_{2}>x_{1}^{2} \bigr\} . $$

If we choose

$$g_{1}(x)=x_{2}-x_{1}^{2}, $$

then

$$g_{1x_{1}}=2x_{1}, \qquad g_{1x_{2}}=1,\qquad \Delta g=2. $$

On $\partial\varOmega_{2}$, $x_{2}=x_{1}^{2}$, then

$$\Delta g_{1}+|\nabla g_{1}|=4x_{1}^{2}+1=4x_{2}+1>0. $$

Then

5.5 $$ \varSigma_{g_{1}}=\partial\varOmega_{2}. $$

If we choose $g_{2}(x)=e^{x_{2}-x_{1}^{2}}-1$, then

$$\begin{aligned}& \frac{\partial g_{2}}{\partial x_{1}}=e^{x_{2}-x_{1}^{2}}(-2x_{1}), \qquad \frac {\partial g_{2}}{\partial x_{2}}=e^{x_{2}-x_{1}^{2}}, \\& |\nabla g_{2}|=e^{x_{2}-x_{1}^{2}}\sqrt{4x_{1}^{2}+1}, \\& \Delta g_{2}=e^{x_{2}-x_{1}^{2}}\bigl(4x_{1}^{2}-1 \bigr). \end{aligned}$$

Thus on $\partial\varOmega_{2}$,

$$\Delta g_{2}+|\nabla g_{2}|=e^{x_{2}-x_{1}^{2}} \Bigl(4x_{1}^{2}-1+\sqrt {4x_{1}^{2}+1} \Bigr)=e^{x_{2}-x_{1}^{2}}(4x_{2}-1+\sqrt{4x_{2}+1}), $$

which implies that

$$\varSigma_{g_{2}}= \biggl\{ x\in\partial\varOmega_{2}: x_{2}\leq\frac {3}{4} \biggr\} \subset\partial \varOmega_{2}. $$

If we choose $g_{3}(x)=\ln(1+x_{2}-x_{1}^{2})$, then

$$g_{3x_{1}}=\frac{-2x_{1}}{1+x_{2}-x_{1}^{2}},\qquad g_{3x_{2}}= \frac{1}{1+x_{2}-x_{1}^{2}}, $$

and

5.6 $$\begin{aligned} \Delta g_{3}&=-2\frac{(1+x_{2}-x_{1}^{2})+2x_{1}^{2}}{(1+x_{2}-x_{1}^{2})^{2}}-\frac {1}{(1+x_{2}-x_{1}^{2})^{2}} \\ &=-\frac{2(x_{2}+x_{1}^{2})+3}{(1+x_{2}-x_{1}^{2})^{2}}. \end{aligned}$$

On $\partial\varOmega_{2}$, $x_{2}=x_{1}^{2}$, then

$$\Delta g_{3}+|\nabla g_{3}|=-2< 0, $$

which means that there is not boundary value condition (1.6) required, $g_{3}$ is the best general characteristic function.

The second one is the half space $\varOmega_{3}= \{x\in\mathbb{R}^{N}: x_{N}>0 \}$. If one chooses

$$g_{4}(x)= x_{N}^{2}, $$

then

$$\Delta g_{4}+|\nabla g_{4}|>0 $$

and

$$\varSigma_{g_{4}}=\partial\varOmega_{3}. $$

If one chooses

$$g_{5}=x_{N}(1-X_{N}), \quad x_{N}\in \biggl(-1, \frac{1}{2} \biggr), $$

and appropriately defined elsewhere, then

$$\Delta g_{5}+|\nabla g_{5}|< 0 $$

and

$$\varSigma_{g_{5}}=\emptyset, $$

which means that there is no boundary value condition (1.6) required. $g_{5}$ is the best general characteristic function of $\varOmega_{3}$.

## Conclusion

The partial homogeneous boundary value condition no doubt maintain the dominance of this paper. It is a possible abstract application with no physical (trivial) interpretation at this stage. This seems to be good math/science. However, if we regard Eq. (1.1) as a nonlinear heat conduction equation, then the condition (1.6)

$$u(x,t)=0,\quad (x,t)\in \varSigma_{g}\times(0,T), $$

implies that on this part we must control its heat conduction by technical means. No boundary value being imposed on $\partial\varOmega \setminus\varSigma_{p}$ implies that there is a thermal insulation on $\partial\varOmega\setminus\varSigma_{p}$, the heat conduction cannot pass $\partial\varOmega\setminus\varSigma_{p}$.

For a parabolic–hyperbolic equation, how to impose a suitable partial boundary value condition to ensure the well-posedness of the entropy solutions is a very interesting problem. This problem can be traced back to 1960s, which is called the theory of the second order differential equations with nonnegative characteristic form. In brief, for a degenerate elliptic (or parabolic) equation, the partial boundary on which the boundary value should be imposed is determined by the diffusion coefficient $a^{rs}$, the Fichera function $b_{r}-a^{rs}_{x_{s}}$ and the inner normal vector $n=\{n_{r}\}$. When the equation becomes nonlinear, for example, in the equation considered in this paper, there is no diffusion coefficient, it is almost impossible to find a function similar to the Fichera function to express the boundary value condition. In this paper, a new method—the general characteristic function method—is introduced. Instead of the Fichera function, the partial boundary on which the boundary value should be imposed, can be expressed by the general characteristic function. One can see that the partial boundary value condition (1.6) with the expression of $\varSigma_{p}$ (1.7) changes along with the choice of the general characteristic function. The simplest one is the distance function from the boundary $d(x)=\operatorname{dist}(x, \partial\varOmega)$, in this case, $\nabla d =n$ is the inner normal vector of *Ω*. The novelty of this method lies in that there is not any requirement of the regularity of the weak solutions on the boundary, and it can be generalized to the other kinds of the degenerate parabolic equations. A fly in the ointment is that it is difficult to find the best characteristic function $g_{0}$ to ensure the best partial boundary value condition. By the way, the domain considered in this paper is unbounded, some innovative techniques are used and can be generalized to the other kinds of the degenerate parabolic equations.
